# Selected Aspects of Self-Regulation: How People Cope with Danger and Change in the Context of COVID-19 (Research in Poland and Ukraine)

**DOI:** 10.3390/ijerph23050606

**Published:** 2026-05-04

**Authors:** Mirosława Huflejt-Łukasik, Anna Szuster, Maciej Pastwa, Adrianna Wielgopolan, Dorota Karwowska, Inna Haletska, Maryna Klimanska, Kamil Imbir

**Affiliations:** 1 Faculty of Psychology, University of Warsaw, Banacha 2D, 02-097 Warsaw, Polandkimbir@psych.uw.edu.pl (K.I.); 2 Department of Humanities and Social Sciences, WSB Merito University in Gdańsk, Al. Grunwaldzka 238A, 80-266 Gdańsk, Poland; 3 Department of Psychology, Ivan Franko National University of Lviv, Doroshenka Str. 41, 79000 Lviv, Ukraine; marina.klimanska@gmail.com

**Keywords:** self-regulation, danger, COVID-19 pandemic, emotions, protective mechanisms

## Abstract

**Highlights:**

**Public health relevance—How does this work relate to a public health issue?**

- The study identifies psychological mechanisms influencing adaptation to prolonged public health crises, using the COVID-19 pandemic as a naturalistic high-threat context.
- It distinguishes two types of negative emotions that play different roles in shaping individuals’ responses to danger, informing how people cope during widespread health threats.

**Public health significance—Why is this work of significance to public health?**

- The findings show that protective self-regulatory resources like goal congruence and a positive future self are associated with lower perceived danger and mitigate the impact of negative emotions during a pandemic.
- The model demonstrates consistent mechanisms across time and cultural contexts (Polish and Ukrainian samples), indicating universal psychological processes relevant to population-wide health emergencies.

**Public health implications—What are the key implications or messages for practitioners, policy makers and/or researchers in public health?**

- Strengthening individuals’ sense of future orientation and continuity of goals may be associated with greater resilience and with lower perceived danger during major health crises.
- Public health interventions and communication can benefit from incorporating strategies that support self-regulatory resources, improving psychological adaptation in high-uncertainty environments.

**Abstract:**

This study examines how individuals adapt to situations of danger and change arising from the COVID-19 pandemic by testing a model based on self-regulatory mechanisms. The model includes three key elements: (1) the intensity of negative emotions as a manifestation of affective reaction to situations of danger and change and, at the same time, as a signal activating self-regulatory processes; (2) protective mechanisms in change, that is, the perception of goal congruence and a positive future self; and (3) the individual sense of danger and the overall sense of danger at various physical distances to the self. The study was conducted online. Participants completed a set of standardized questionnaires assessing negative emotions, perceived danger, and protective mechanisms across three measurement waves corresponding to successive stages of the COVID-19 pandemic. The model was validated in three waves of the pandemic in Poland and one in Ukraine. To verify the relationships between the measured variables, a structural equation model was constructed using the RStudio software (version 1.2, 2019) with the Lavaan package. The results were consistent with the prediction model, which considers the relationships between the intensity of negative emotions and the intensity of protective mechanisms in change and perceived danger. Two factors within negative emotions were identified, each interacting differently with self-regulation. Negative emotions were related to the intensity of perceived danger, while protective mechanisms were linked to the reduction in danger. The results confirm the complex nature of self-regulation mechanisms both affectively and cognitively and also their orientation toward the future, which constitutes a protective resource. The study’s limitations include the online method, which limits standardization, control, and the sample’s limited randomness and inequivalence of measurements and sampling in Ukraine and Poland.

## 1. Theoretical Introduction

The aim of the presented research was to explore selected self-regulation mechanisms and the relationships between them, activated in the face of a long-term, global danger that was the COVID-19 pandemic in Polish and Ukrainian conditions.

Adaptive strategies for coping with danger and crisis situations are a result of subjective perception and objective danger. This generates attitudes aimed at restoring a state of safety or preventing its loss. Coping with danger is a dynamic process with a complex structure, generating affective, cognitive, and behavioral actions. Confrontation with a new, threatening situation requires going beyond automatic response strategies, modifying old ones, and generating new behavioral patterns. The complexity of self-regulation mechanisms is expressed in their heterogeneous origin, these being both automatic and reflective as well as conscious and unconscious [1,2,3].

We focused on selected aspects of self-regulation, like (a) the intensity of negative emotions treated as an affective response to danger, simultaneously activating self-regulation processes; (b) protective mechanisms in change: perceived goal congruence (before and after change) and positive future self; and (c) the individual sense of danger and the overall sense of danger at various physical distances to the self.

### 1.1. Self-Regulation Mechanisms and Emotions as a Response to Danger

The concept of self-regulation includes various mechanisms allowing an individual to control their own functioning, regulate activity, current state, and internal processes [1]. In the context of danger, the ability to control stress responses, maintain attention, and interpret one’s own internal states and those of others is important [4,5].

“Emotions are one of the key psychophysiological processes that help people adapt to the environment and achieve their goals” [6,7]. They lead to changes in attention, activation of association networks, changes in response hierarchy, and rapid integration of changes in the autonomic nervous system, muscle tension, and facial expressions. These coordinated psychological and physiological changes generate a coherent response that enables adaptation and effective performance at both interpersonal and intrapersonal levels [8]. The functionality of emotions is a fundamental property [9]. There is a strong temptation to treat all emotions as if they all perform the same functions. However, it is important to consider the differences, the variation in contexts in which they appear, and how the individual frames the experience that is the emotion [9,10].

The intensity of a given emotion is one of its most characteristic features. It exerts an influence on people’s motivation and behavior. Emotional intensity is reflected in subjective experience, but also in the form of mimic, visceral, and nervous reactions [7,11]. Just like in the case of the emotion itself, its duration is limited. Variations on the emotional activity scale are regarded as relatively independent from the type of experienced emotion, its indication (positive or negative), or content [12,13]. What seems to be an important determinant of emotional intensity is the motivational meaning of a given situation and its influence on an individual’s goals [7,14,15,16]. Regulation changes in the emotional process occur mainly at increasing or decreasing intensity, and there are temporal features like the moment the emotion appears or its persistence [17].

Both functionality and intensity aspects mean that, in the context of danger, emotions are one of the signals initiating conscious self-regulation processes. Their dynamics inform about the course of self-regulation, including the need to withdraw and change the direction of attention [18,19,20,21,22]. Although research indicates that the activation of self-regulation (i) does not require the conscious experience of emotions [23], greater intensity and clarity of experienced emotional states serve to undertake action [20,22] and promote effective self-regulation.

Thus, the emergence of a given emotion and the intensity of its experience in confrontation with the pandemic situation activate the self-regulation processes.

### 1.2. Protective Mechanisms as Self-Regulation Resources

Existence under the conditions of the COVID-19 pandemic was a new situation for contemporary generations, not encountered for decades on a global scale. The previous pandemic (influenza) took place in the years 1917–1918; although the incidence rate was lower (approx. 500 million compared to 770 million in the case of COVID-19), the number of casualties was unprecedentedly greater (50 million compared to 7 million). Its significance, although symbolic, facilitated a negative affective context and intergenerational transmission of a sense of danger [24]. In the face of the contemporary pandemic, we have come to create new or modify old action schemas and goal-achievement strategies.

Conditions of this kind also engage protective mechanisms concerning situations of change [25,26,27,28,29,30,31,32]. Results of research on the changes have allowed the identification of mechanisms that buffer the negative effects of change; i.e., they lower distress and the symptoms of psychopathology. We focused on two mechanisms: the congruence of a person’s goals before and after change and the imagination of a positive, future self. The function of protective mechanisms is to limit the negative consequences of change, and they also constitute a specific self-regulation resource [25]. Both goals and the positive future self act as standards in self-regulation processes [33,34]. The self-regulation process itself involves setting goals (standards) and perceiving them as values referring to a feedback system and actions aimed at achieving a given goal [20,21,35]. In the goal hierarchy in self-regulation processes, the highest level is the goal of defining what kind of person one wants to be (so-called self-guides; cf. [36,37,38]). Goals from subsequent levels concern specific physical actions and behaviors [21,22]. Thus, a positive future self, as well as greater congruence of a person’s goals (before and after change), are characteristics of the constructiveness of goals activated during an individual’s self-regulation.

#### 1.2.1. Positive Future Self as a Self-Regulation Resource

Experienced change, regardless of whether it is perceived as positive or negative, is associated with negative emotions [39,40]. The negative consequences of perceived change (positive and negative) are smaller in individuals characterized by a sense of self-stability [32,41]. Despite changes in self-content during life and under the influence of experiences [42], only some aspects of the self are particularly susceptible. This applies to the actual self [28,37,43]. The specificity of such changes in the self-image allows for better adaptation to new situations, and, at the same time, does not inhibit one’s own development. At the same time, content concerning the future self (so-called self-guides, e.g., possible selves, ideal self) stabilizes the sense of identity despite changes in the content of the actual self [26,29,43,44,45,46]. They represent individuals’ imaginations oriented toward the future, constituting a link between cognition and motivation [47,48,49]. They act as incentives for future behavior, providing an evaluative and interpretative context for the current self-view, allowing the protection of one’s own emotions when, for example, the actual self is not perceived positively. Research results confirm the links between the activation of what people are able to do and their achievements [50].

Also, the importance and complexity of possible selves [49], the closeness of the imagined future [51], feasibility [47], and connections between the actual and possible self [48] favor the effectiveness of goal achievement.

The adaptiveness of the positive content of self-standards manifests in directing an individual’s aspirations, building hope, and opening positive perspectives for the future. They contain important developmental standards in the form of a positive self-concept. Research conducted during the COVID-19 pandemic has shown that better adaptation (i.e., adherence to recommendations) is facilitated by communication consistent with personal identity, linking identity with action, and perceiving the crisis as an opportunity for self-improvement (IBM theory) [52,53]. Furthermore, positive contents of the ideal or possible self can balance less positive or even negative contents of the actual self [36,43]. Considering that the assessment of congruence is a mechanism for restoring coherence in the self-structure [54], adding new, positive self-elements potentially changes negative contents that were not directly in the focus of attention and change. This is confirmed by research results concerning psychotherapy or life experiences, in which the positive future self plays a supportive role in self-regulation and personal performance [25,36,47,55,56,57,58,59,60].

The possible self is a unique construct: on the one hand, it stabilizes and protects a person’s self, and on the other, it refers to specific social roles, situations, or contexts in the form of individual images and is spontaneously modified under the influence of life changes [27]. A person can create many positive or negative images about themselves in the future. Also, a change in the situation induces changes in the content of the possible self, which favors adaptation to the new situation [30]. Research on change related to entering a new (fatherhood) role showed that it favors generating new, possible selves related to the situation and influences presented behaviors [27].

Possible selves also have a protective function. Individuals who experienced a crisis and noted its impact on their current life activate both the negative, actual self and possible selves. However, those who perceived the crisis as a past and concluded episode generated positive images of possible self and assessed them as more probable than individuals from the control group who did not experience a crisis [26]. Possible self, on the one hand, stabilizes and supports self-coherence, allowing for a desired imagination of the future, and, on the other hand, modifies the self, contributing to adaptation to a new situation. In the model, we therefore included the positivity of the future possible self as a protective mechanism in changes caused by COVID-19.

#### 1.2.2. Congruence of Personal Goals with the Self as a Source of Self-Regulation

Congruence between goals and the self is associated with higher well-being [61,62]. Results confirm the importance of congruence between the content of the actual self, which has changed, and what the person wanted regarding their own self before the change for perceived well-being [29].

People differ in their ability to adjust the content of their actual self to the content of their self before the change, and, consequently, they differ in their ability to adapt [31]. As data indicate, a higher level of so-called “derailment,” i.e., the level of destabilization of self-concept as a result of change, is associated with an increase in symptoms of depression, anxiety, perceived stress, and reduced well-being [63]. Surprisingly, “derailment” is linked to depressive symptoms regardless of stress level [64]. These researchers also showed that the “derailment” effect decreases when, even during a brief intervention, a person’s sense of goal continuity is strengthened by seeking connections between the earlier self and current goals [31].

Another important aspect emphasized in the context of change research is the role of awareness of a central life purpose, which influences other goals, behaviors, and sense of meaning [41]. The study results indicate that a greater sense of meaning buffers against negative emotions in individuals who do not perceive what has happened in their lives over the last 10 years as positive. A sense of meaning is associated with self-coherence [65,66], which constitutes an element of enhancing well-being in the face of changes. Indeed, a sense of meaning helps moderate temporal differences between perceived current and past self [41]. Individuals going through negative experiences are motivated to seek meaning in their lives, adapt to the situation, and pursue important goals consistent with their own [67]. This justifies including another protective mechanism in our model—the perception of congruence of actual, everyday, and important goals with those prior to the change.

### 1.3. Sense of Threat as a Reaction to the Pandemic Situation

In response to COVID-19, each country undertook its own combination of non-pharmaceutical interventions (NPIs), including resource allocation, risk communication, social distancing, and travel restrictions, which are mainstream actions designed to control the spread of the coronavirus disease [68,69].

These differences in strategies or their combinations in individual countries could translate into differences at the level of individual or group perception of danger. Results of research using the Perceived Stress Scale questionnaire showed that, despite differences, the level of perceived stress during the COVID-19 pandemic was higher [70] than usual in the general population [71]. In the Polish population [72], the values were similar to those reported by Chua et al. [73] during the SARS epidemic in 2003.

Research conducted in Poland during the first phase confirmed that the COVID-19 pandemic was a very stressful event for 75% of participants and the strongest factor predicting adjustment disorders. Increased symptoms of adjustment disorders were reported by 49% of individuals [74]. The COVID-19 pandemic constituted not only a global health crisis (including mental health) but also one of social, interpersonal, and intrapersonal relationships [75]. The sense of danger was the main parameter defining a person’s psychological situation during the pandemic.

Research shows that danger and negative emotions also favor maladaptive manifestations of self-regulation. Their manifestation was an increase in belief in conspiracy theories [76] and the intensification of optimistic cognitive distortions [77,78], which lowered motivation to undertake protective behaviors.

An important source of the sense of threat is the subject’s assessment of the situation. 

The cognitive aspect includes the analysis of the situation’s content, assigning its assessment, but also its potential reanalysis, which may change the initial assessment of the situation as threatening [79]. In confrontation with danger, strategies influencing perception are activated, including devaluation of the threat, unrealistic optimism, avoidance [77], and also positive reframing [80]. The feeling of danger may also be related to the physical location.

A person’s location has a unique and distinguished psychological character. One’s own property, like territory [81], place, culture [82], or social status [83], is perceived differently and generally more positively valued. This shows the importance of physical and psychological distance for perception and evaluation [84,85] and also the dominant role of the self in generating egocentric distortions [26,86,87]. These may manifest in the form of asymmetry and differences in the assessment of the risk of one’s own and others’ situations. While one’s own is assessed as more favorable, what is at a greater physical distance from the self and concerns others is assessed as a greater danger. Asymmetric assessment of one’s own situation and situations physically distant from the self has also been noted in other studies on COVID-19 [77,78,88].

At the same time, research results confirm an increase in the general sense of danger [70,77], so the global sense of danger (regardless of distance from the self) should be higher. Therefore, when investigating, we differentiated its manifestations and measured (a) individual sense of danger and (b) overall sense of danger at various physical distances to the self (the country where the subject lives, Europe, and the world).

### 1.4. Current Research

Research, identification, and understanding how people cope and adapt to an unpredictable situation that carries danger and significant life changes seems to be a particularly important task [89] and crucial for public health and social sciences.

The results of the research cited above indicate the specificity of the pandemic’s course and reactions to it in different countries. Therefore, the applicability of the proposed model is limited to Polish and Ukrainian conditions, although currently the pandemic is considered a fairly homogeneous crisis despite local differences [90].

The presented model contains psychological dimensions also present in other models of coping with stress, like the cognitive-transactional model. This model emphasizes the continuity of cognitive and behavioral coping with external and internal demands that are assessed as excessive or overwhelming [91]. Our proposal has a more general dimension. It is not focused on identifying a style of coping with stress (emotion-focused, problem-focused, or meaning-focused) or avoidance [92]. It is about capturing what happens in the area of self-regulation.

The aim is to capture and describe the psychological mechanisms activated in the face of the COVID-19 pandemic.

We assumed that negative emotions and their intensity constitute the starting point of the model based on knowledge of the functions of emotions (cf. introduction), The focus on the negative valence of emotions was justified by (1) the global, negative evaluation of pandemic conditions worldwide, (2) their dominant significance in self-regulation processes, and (3) empirical reports dominated by indications of manifestations of negative affective reactions and their intensity [77,93,94].

Emotions and their intensity are a signal activating motivational processes, which in turn initiate self-regulation processes. Although people differ in how intensely they experience emotions, it seems that under conditions of a global crisis such as the pandemic, the variance in the intensity of experienced emotions was significantly smaller, and globally their level was significantly higher [74,75].

Since the pandemic was also a period of important change in the lives of individuals and societies, the model included two protective mechanisms: positive future self and perceived congruence of goals before and after change [25]. The premise for their inclusion is that new situations, besides strong emotions, are also signals eliciting self-regulation. We verified the relationships between the intensity of negative emotions, protective mechanisms in change, and perceived danger.

#### Hypotheses and Theoretical Model

In the model, we included selected elements of self-regulation activated in situations of danger and change. We focused on the relationships between (a) experiencing negative emotions as an affective reaction to danger, (b) protective mechanisms in change (positive future self and goal congruence), and (c) perceived danger in the form of an individual’s subjective sense of danger and the overall sense of danger at various physical distances to the self.

**Figure 1:**
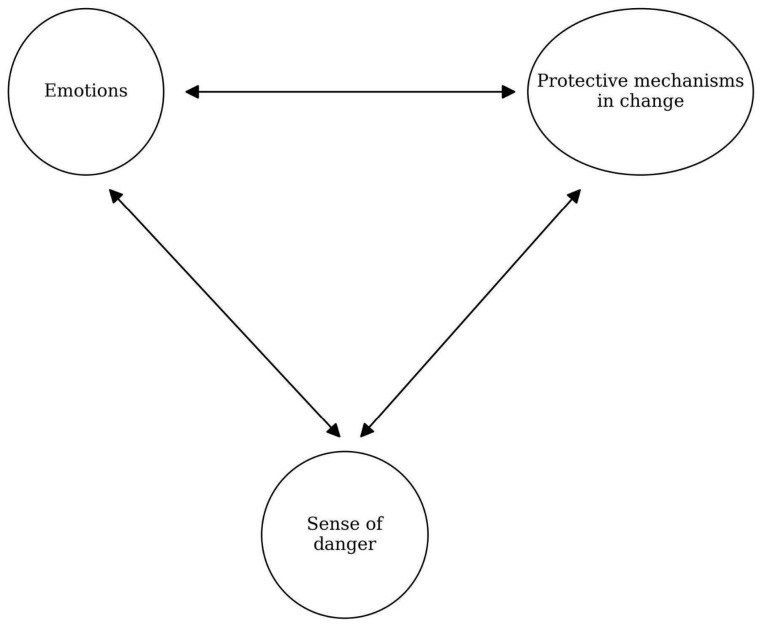
Research variables.

**Figure 2:**
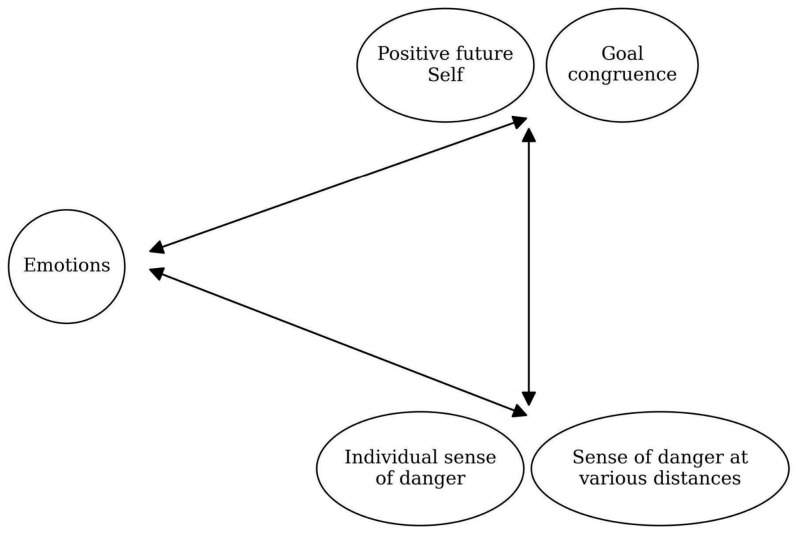
Research variables in detail.

The model describes the intensity of negative emotions on protective mechanisms in change and perceived danger (see Figure 1 and Figure 2). Negative emotions would be associated with higher levels of perceived danger and lower levels of protective mechanisms (Figure 1).

As for the role of protective mechanisms—positive future self and the congruence of a person’s goals before and after change—we expected that their presence would be associated with a less intense sense of danger. The greater the intensity of the positive future self, the lower the intensity of negative emotions and the smaller the intensity of the sense of danger. The premises for such expectations are the results of research [25,26], indicating that a positive future self can buffer the negative effects of changes, including those concerning negative emotions.

Cognitive appraisal of danger generates different affective consequences depending on how the subject perceives the situation and what beliefs they have regarding its consequences in the form of positive or negative consequences for the individual [39,40]. Assessing the situation as carrying negative effects is associated with negative consequences for a person’s health and emotions. On the other hand, assessing change as positive limits negative effects in the area of psychosomatic symptoms and also favors coherence, clarity of self-image, and the subject’s well-being [36].

Furthermore, we expect a negative relationship between goal congruence and the intensity of negative emotions and sense of danger: the greater the congruence of goals before and after change, the lower the intensity of negative emotions and sense of danger [31]. A sense of continuity at the level of goals, on the one hand, is associated with subjectively perceived stability of one’s own situation and self-image [25,36] and, on the other, limits the impact of potential change, allowing for relative invariance of daily functioning.

We verified the model over time by examining it at different stages of the pandemic in Poland and also in Ukraine.

## 2. Method

The study presented in the current manuscript was a part of a larger research project; some results from the project were presented in an already published article [77]. The methods of collecting samples and the measures presented in the current article are highly similar or the same as those in the previous text, which is addressed in the appropriate sections of the current article. The pandemic situation in Poland and Ukraine during the study, setting the context for the research, is described in the Supplementary Material S1.

### 2.1. Participants

#### 2.1.1. Poland—Stage 0

The participants in this early, exploratory stage of study were volunteers recruited on social media (Facebook) groups related to the university or local communities. The participants did not receive remuneration for taking part in this stage of the study. The survey was conducted using the Qualtrics online interface, and the participants could enter the survey only once. The sample at Stage 0 consisted of 1202 participants, including 986 women, 211 men, and 5 research subjects who described their gender as other. The participants were aged from 18 to 78 years old (M = 35.05; SD = 12.81). The proportion of men to women among participants in Stage 0 was 0.22, while in the Polish population it was 0.94 in 2020. The sample had a much smaller representation of men than women compared with the population. The median age of Polish citizens in 2020 was 41.9 years of age, which is 6.85 years older on average than the mean age in the Stage 0 sample [95].

#### 2.1.2. Poland—Stages 1 and 2

Stages 1 and 2 of the study were conducted using the Ariadna research panel, an online research interface similar to Amazon, mTurk, or Prolific. It allows the preparation and sharing of surveys, as well as recruiting participants using various methods like social media, mailings, or the snowball method. The participants in these stages received remuneration for their participation in the form of points, which they could exchange for products in the research panel.

Stages 1 and 2 were conducted using the repeated measures method, using the same sample in both stages. However, it is common in studies using this method that some of the participants drop out between stages of the study. Therefore, the final samples consisted of 1179 participants in Stage 1 (586 women, 593 men) and 1014 participants in Stage 2 (488 women, 526 men). The participants in both samples were aged from 18 to 85 years of age, with the following descriptive statistics: Stage 1, M = 44.47, SD = 15.77; Stage 2, M = 45.72, SD = 15.40. The proportion of men to women among participants for Stage 1 was 1.01 and 1.08 for Stage 2, which means that in both stages, men were overrepresented when compared to the proportions in the Polish population. In Stage 1, the mean age of participants was 2.57 years older than the median in the Polish population, while in Stage 2, it was 3.82 years older.

The methods of collecting the Polish samples were the same as in Szuster et al. [77]; however, different methods of filtering the data were employed for the current study, as more measures were used for the analyses.

#### 2.1.3. Ukrainian Sample

The participants taking part in the measurement conducted on the Ukrainian sample were volunteers who did not receive remuneration for their participation. Since the survey was conducted using the Qualtrics online interface, the participants could enter the survey only once. They were recruited from various social media (Facebook) groups related to the university or local communities. The sample consisted of 1003 participants, including 800 women and 203 men. The participants were from 18 to 76 years of age (M = 36.42; SD = 12.27). The proportion of men to women in the Ukrainian sample was 0.25, while in the Ukrainian population in the year 2020, it was 0.87; the sample had a much smaller representation of men than women compared with the population. The median age of Ukrainian citizens in the year 2020 was 41.2 years of age, which is 4.78 years older on average than the mean age in the Stage 0 sample [96].

### 2.2. Measurements

#### 2.2.1. Emotions

The intensity of 20 negative emotions was measured using the following indirect question: “Assess to which extent emotions given below cause people to experience danger in the current situation”. The emotion measure captured the perceived emotional contribution to threat appraisal, rather than direct affective intensity. The validity of using this indirect method of measuring emotions was verified in a pilot study and is described earlier in an article alongside the rationale for picking these particular 20 emotions [77]. The pilot study showed that both the declared intensity and variance were higher for emotions measured with an indirect method rather than a direct one. The measured emotions were: suffering, helplessness, frustration, breakdown, terror, bitterness, aversion, disgust, abhorrence, repulsion, humiliation, shame, embarrassment, disappointment, disillusionment, sadness, sorrow, depression, envy, and contempt. The emotions were presented in a random order for each participant. The participants from the Polish sample in Stage 0 stated their answers on a scale from 1 (“to a small extent”) to 100 (“to a high extent”), and the participants from the Ukrainian sample and Polish sample from Stages 1 and 2 answered on a scale from 1 (“to a small extent”) to 7 (“to a high extent”). We divided the measured emotions into two emotional factors used in the analyses, namely, Factor 1: sorrow, helplessness, sadness, breakdown, depression, frustration, terror, suffering, disappointment, disillusionment, and bitterness (11 emotions); and Factor 2: contempt, disgust, aversion, abhorrence, repulsion, shame, embarrassment, and envy (8 emotions). Humiliation was excluded from further analyses. The exploratory factor analysis, constituting the factors, as well as confirmatory analyses for the factors for all stages of the study, are described in the Supplementary Material S2.

#### 2.2.2. Protective Mechanisms: Positive Future Self and Goal Congruence

Protective mechanisms in the face of the pandemic were measured in all samples with two variables. The first was a question about the possibility of perceiving a positive perspective of self, namely, “To what extent are you able to imagine yourself and your positive future?” with answers on a scale from 1 (“I am not able at all”) to 7 (“I am definitely able to”). The second variable was related to congruence with one’s personal goals before and after the change/pandemic and consisted of two questions: “To what extent are you able to achieve your everyday work and personal goals now?” and “To what extent are you able to achieve goals and values important for you that originate from before the epidemic?”, with answers on a scale from 1 (“I am not able at all”) to 7 (“I am definitely able to”). Correlations between these two questions are described in the Supplementary Material S2. The measurement index is the sum of the answers to all questions.

#### 2.2.3. Sense of Danger: Individual Sense of Danger and the Sense of Danger at Various Distances

Two variables were used with respect to the sense of danger perceived by the participants. Individual sense of danger was measured with three questions: “Considering the current situation, to what extent do you feel threatened physically (by pain, afflictions, disease)?” “To what extent do you feel threatened mentally (by worries, anxiety, fear, panic)?” and “To what extent do you feel threatened by the dynamics of the epidemic (by getting infected)?”. The participants were answering on a scale from 1 (“I do not feel worried at all”) to 7 (“I definitely feel worried”). The second variable was related to the sense of danger at various distances; the results regarding this measure were described independently in our previous paper [77]. The three questions measuring the perceived danger at different distances were: “To what extent do you feel threatened by the epidemic situation in Poland?”, “To what extent do you feel threatened by the epidemic situation in Europe?” and “To what extent do you feel threatened by the epidemic situation around the world?”. Participants answered on a scale from 1 (“I feel threatened to a small extent”) to 7 (“I feel threatened to a large extent”). The measurement index is the sum of the answers to all questions. Cronbach’s alphas for both variables for each of the stages are presented in Supplementary Material S2.

### 2.3. Procedure

The study was conducted on three separate samples, two Polish and one Ukrainian, with three consecutive measurements on one of the Polish samples. The study was conducted in 2020, and the exact dates of the measurements were: Stage 0 (Polish sample): 19–24 March; Ukrainian sample: 8–30 April; Stage 1 (Polish sample): 4–7 May; and Stage 2 (Polish sample): 4–17 June.

Online questionnaires were used in the study, and participants could enter the questionnaires only once. At each stage of the study, participants gave their written consent by answering the question in the survey. All participants answered the same questions described above; however, the order of the questions differed between stages. In Stage 0 and in the Ukrainian sample, participants first answered questions regarding individual sense of danger, then the questions regarding emotions, followed by the questions regarding the perspective of self and achieving goals, the questions regarding sense of danger at different distances, and with the questions regarding demographics at the end. For stages 1 and 2, demographics were declared by the participants at the beginning, then they assessed the emotions felt in the face of the pandemic, next they answered questions regarding individual sense of threat, followed by the questions regarding perspective of self and achieving goals, and then the questions regarding danger at different distances at the end. The questions about demographics were about participants’ sex and age. The study was a part of a larger research project on the COVID-19 pandemic conducted at the Faculty of Psychology, University of Warsaw; the procedure of the study is highly similar to the one described in Szuster et al. [77].

The study design and procedure were approved by the bioethical committee of the Faculty of Psychology, University of Warsaw. All procedures involving human participants were conducted in accordance with the ethical standards of the institutional and/or national research committee and with the 1964 Helsinki Declaration and its later amendments or comparable ethical standards [97].

### 2.4. Transparency and Openness

We report data exclusions and all measures in the study, and we follow JARS [98]. All data for this study are available at the following link: https://figshare.com/s/615f0349078e2c79ec02 (accessed on 12 of March 2026). The databases shared in the repository contain only the data collected for the presented research. The research we conducted was part of a larger study examining the functioning and psychological well-being of people in Poland during the pandemic. All the data collected in the wider project is not shared in the repository for this article. Translation of all research materials can be found in the Supplementary Material S3; the questionnaires in exact Polish wording may be shared after a reasonable request. Data were analyzed using IBM SPSS Statistics 29 (descriptive statistics) and RStudio (testing hypotheses) software. This study’s design and its analysis were not pre-registered, as the study was conducted during the outbreak of the pandemic, and we wanted to register the initial psychological reaction.

## 3. Results

In order to verify the relations between the measured variables, a structural equation model was constructed using the RStudio software [99] with the Lavaan package version 0.6-19 [100]. The model was established based on the data from the Polish sample, Stage 0, then verified on the data from Polish samples Stage 1 and 2 and the Ukrainian sample. We put the sets of variables from each sample separately, building one model at a time (as we present in the subsequent sections) as regression models. Every time, we only used the responses from our participants that were complete (there was no missing data; if the response was not finished, it was deleted from the database before analyzing, either by our team or by panel administration before providing the database).

### 3.1. Stage 0

**Figure 3:**
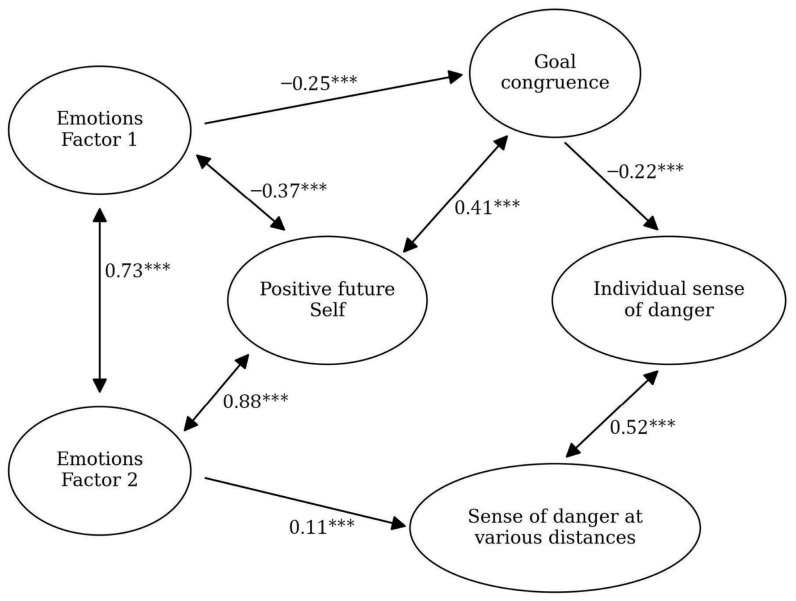
Model for Stage 0 of research conducted on the Polish sample. The black arrows show the relationships between the variables (with standardized beta values written above them and significance levels marked with asterisks: *** *p* < 0.05).

The initial structural model was constructed based on the data from the Polish sample, Stage 0; we have put the entire set of variables in the model, namely the Emotions Factor 1, Emotions Factor 2, positive future self, goal congruence, individual sense of danger, and sense of danger at various distances (as an averaged coefficient from three questions, c.f. Method). The model turned out to be significant: CFI = 0.91, RMSEA = 0.04, *p* = 0.01. In the Supplementary Material S4 we present the descriptive statistics for the variables used in the model (Table S4), and Figure 3 presents the model itself.

### 3.2. Stage 1

**Figure 4:**
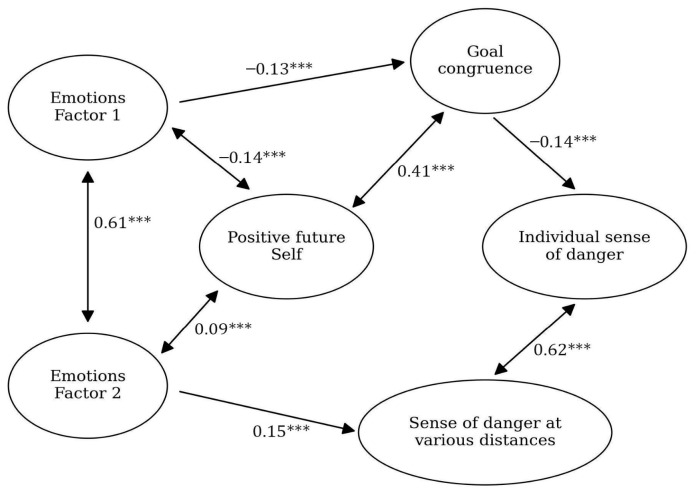
Model for Stage 1 of research conducted on the Polish sample. The black arrows show the relationships between the variables (with standardized beta values written above them and significance levels marked with asterisks: *** *p* < 0.05).

The model from Stage 0 was verified on the data from Stage 1; it turned out to be significant, with a satisfactory fit: CFI = 0.93, RMSEA = 0.05, *p* = 0.03. In the Supplementary Material S4 we present descriptive statistics for the variables used in the model (Table S5), and Figure 4 presents the model itself.

### 3.3. Stage 2

**Figure 5:**
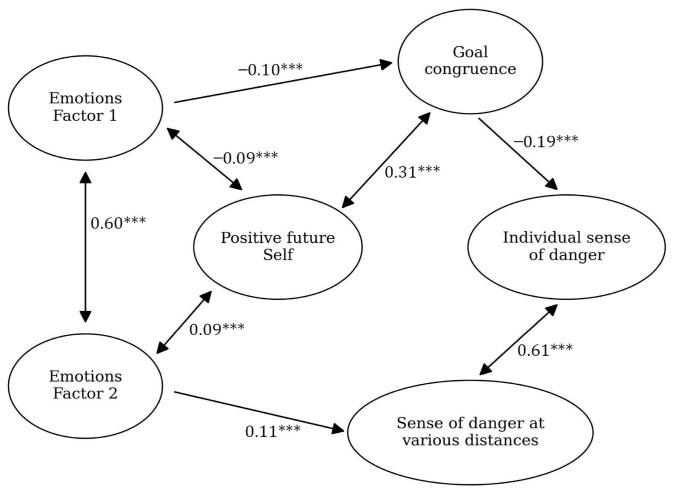
Model for Stage 2 of research conducted on the Polish sample. The black arrows show the relationships between the variables (with standardized beta values written above them and significance levels marked with asterisks: *** *p* < 0.05).

The model was further verified on the data from Stage 2, and again it turned out to be significant, with a satisfactory fit: CFI = 0.91, RMSEA = 0.06, *p* = 0.04. In the Supplementary Materials S4 we present descriptive statistics for the variables used in the model (Table S6), and Figure 5 presents the model itself.

### 3.4. Ukrainian Sample

**Figure 6:**
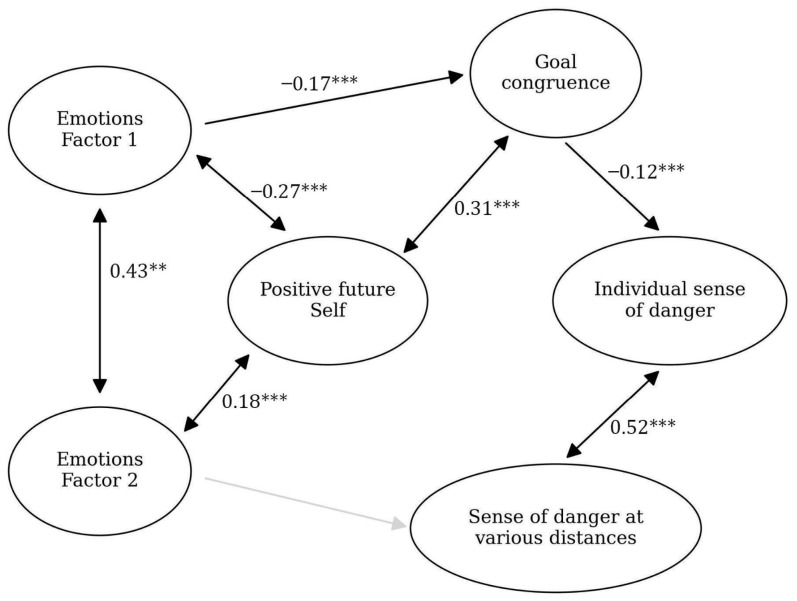
Model for the Ukrainian sample. The black arrows show the relationships between the variables (with standardized beta values written above them and significance levels marked with asterisks: ** *p* < 0.01, *** *p* < 0.05). The gray line shows an insignificant relationship.

The model was further verified on the data from the Ukrainian sample. It turned out to be significant, with a satisfactory fit: CFI = 0.95, RMSEA = 0.03, *p* = 0.02. In the Supplementary Materials S4 we present descriptive statistics for the variables used in the model (Table S7), and Figure 6 presents the model itself.

## 4. Discussion

The aim of the study was to search for links between selected aspects of self-regulation to identify the response mechanisms to danger and change in the context of the COVID-19 pandemic. The starting point for the proposed model was the theoretical assumptions underlying self-regulatory processes.

This exploratory study with a longitudinal design included measurements in 3 phases differentiated by the course of the pandemic. The study also included differences in cultural context within a shared macro-regional framework. It included three measurements in the Polish sample and one measurement in the Ukrainian sample.

The results were consistent with the predictions of the model: negative emotions were shown to be associated with higher levels of perceived danger, whereas protective mechanisms were associated with lower levels of perceived danger.

### 4.1. The Significance of Emotions and Self-Regulation

Factor analysis applied to negative emotions revealed two factors that were strongly positively related. Both types of emotion were included as subvariables and included the emotional states described below.

Emotions 1—these are sorrow, helplessness, sadness, breakdown, depression, frustration, terror, suffering, disappointment, disillusionment, and bitterness, i.e., emotions directed at the self that are an expression of one’s own state and characterized by low arousal. They can be considered to be linked to the process of self-regulation and self-focused attention as one of the phases of self-regulation [20,21]. In this context, they are a signal of either the necessity to activate self-regulatory processes or of the ineffectiveness of those processes. Emotions 2—these include contempt, disgust, aversion, abhorrence, repulsion, shame, embarrassment, and envy, i.e., linked to relations with others, how others are perceived, and also being avoided. They can also be seen as a self-regulation trigger signal, but focused on one’s own person. They are more for comparison with others to create one’s own positive self.

The consistent emergence of this two-factor structure across waves and in both countries indicates that emotional responses to prolonged danger were selective rather than undifferentiated. At the same time, the strong correlation between both emotion types suggests that they co-occurred within a shared affective field shaped by the pandemic context.

### 4.2. Emotions, Protective Mechanisms, and Self-Regulation

Higher intensity of self-focused emotions is associated with a less positive future self and lower goal congruence. At the same time, a positive future self is negatively related to the intensity of these emotions. These relationships suggest the destructive role of passive, self-centered negative emotions for self-regulation. They may also indicate that individuals became more aware of the negativity of their situation. Furthermore, the results demonstrate the protective role of a positive future self.

The pattern of relationships between the second category of emotions and other aspects of self-regulation is different: emotions oriented toward the external situation, including others, were strongly and positively related to a positive future self. It seems that negative emotions perceived in relation to others may allow individuals to distinguish the self in a more positive way.

This picture of relations can also be interpreted in terms of relevance. The deferred interplay between the identified emotion factors and the different aspects of self-regulation confirms it is valid to distinguish them clearly.

The picture of emotions and protective mechanisms revealed and replicated in subsequent study phases shows both positive and negative relations that are both bilateral and unilateral. The bilateral indicates the interplay of factors, as in the case of the relationship between negative emotions and the positive future self (although the sign of these relationships differed). In contrast, the one-sided relationship between the intensity of negative emotions included in factor 1 (passive focusing on self) and goal congruence shows that the lower the intensity of passive negative emotions, the higher the goal congruence before and after change; thus, the affective factor is a predictor of goal congruence. This pattern highlights the functional heterogeneity of negative emotions in situations of chronic, socially mediated danger.

### 4.3. Emotions, Protective Mechanisms, and Perceived Danger

Results from all measures indicate protective mechanisms are at work, albeit in varying degrees of intensity. Higher goal congruence is related to reduced levels of threat. And in the case of a positive future self, the impact occurred through goal congruence. With the interplay of protective mechanisms, the strength of the relationship was fairly constant across all study measurements.

The differentiation of negative emotions turned out to be important for threat assessment: only type 2 negative avoidance emotions directly increased the assessment of danger at various distances to the self (own country, Europe, and the world), and this in turn increased the individual’s sense of danger. The two aspects of perceived danger interacted quite strongly with each other. This result applied to studies in Poland at different stages of the pandemic. This dependency model was reproduced in the study conducted in Ukraine, with one exception: it did not reveal a direct link between negative emotions and risk assessment in various locations at different physical distances to the self. Perhaps Ukraine’s lack of membership in NATO and EU structures modified the perception of the threat state, taking into account the location and distance from the self.

The model indicates that cognitive appraisal of danger is mediated by affective reactivity and protective mechanisms by goal congruence and a positive future self. In addition, the subjective appraisal of the individual sense of danger is not directly related to the intensity of the emotion experienced but is mediated (negatively related) through goal congruence, and it is only goal congruence that is negatively related to the appraisal of the individual sense of danger.

### 4.4. Temporal and Contextual Differences

The partial correlations in the individual waves show the results of the tests carried out are consistent with the model and its correlations.

The results from the different waves of the Polish survey are consistent with the model regularities and the results described above. There are differences in the value and strength of associations, especially when looking at wave 0 at the start of the pandemic in Poland and the results of subsequent waves. This may be due to the different method of measurement as well as its timing. Surveys in wave 0 were conducted at the very beginning of the pandemic when the subjective level of threat was the highest [77].

Data collection in Ukraine took place already during the pandemic and corresponded to wave 0 in Poland. And again, the correlations and interactions between emotions, protective mechanisms, and perceived danger were generally confirmed. However, there is no correlation between Type 2 emotions that are oriented towards others and danger assessment at various distances.

## 5. Conclusions

The results obtained confirmed the multidimensional and nuanced nature of the psychological mechanisms activated in situations of danger. They confirm the dynamic nature of the process of self-regulation. The revealed relationships are repeated in subsequent phases of the pandemic and illustrate positive, negative, as well as bilateral and unilateral dependencies. Knowledge of these relations can have an entirely practical and therapeutic sense, pointing to what enhances the self-regulatory potential and allows the person’s relative well-being to be restored. From this perspective, goal congruence and the positive future self have a buffering effect, being linked to the intensity of experienced negative emotions in situations of danger and change. Research in the field of change indicates that even short reflective interventions focusing on goal congruence before and after change and on shaping a positive future self can effectively serve protective functions in the process of self-regulation during change. In this context, the proposed model appears to be a promising framework for understanding and supporting adaptive self-regulation through targeted interventions.

Furthermore, a link between the two types of danger was revealed: the individual sense of danger appeared to be strongly related to the sense of danger at various places (one’s own country, Europe, and the world). This shows the ease with which a sense of danger can generalize to different aspects of a situation: the greater the cognitive evaluation of global danger, the greater the tendency to perceive different places as more threatening, regardless of their specificity and connection to the self.

The relationships between the elements of self-regulation included in the model appear to show a degree of stability over time and across samples: Polish and Ukrainian. They were replicated over time (in the Polish samples), and changes were observed primarily in the strength of associations rather than in their relevance. This pattern points to a certain stability in the psychological processes of self-regulation in response to danger and change.

The question of the universality of the disclosed processes remains an open question and requires further verification in culturally and socially more diverse populations, as well as in contexts involving other types of global threats and large-scale change.

## 6. Limitations

The survey, and consequently the results obtained, has its limitations. One deficit is the single survey on the Ukrainian sample. Having a repeated survey with this sample would firstly balance the scheme, and secondly, it would increase the validity of the interpretation of the model in terms of replicability. At the same time, the proximity of the Polish and Ukrainian cultural contexts (due to physical distance as well as historical commonality) limits the possibility of interpreting universality.

The online setting of the study and the use of different platforms for conducting the research (a professional research panel in Poland and various social media groups on Facebook in Ukraine) mean that the samples are not collected according to identical criteria in both countries. They are not representative of the population of the countries they originate from. This could be seen when the age and gender structure of the samples is compared with the respective structures for Poland and Ukraine. These discrepancies limit the possibility for generalization of the results. However, conducting the surveys at the beginning of the pandemic provided the unique opportunity to observe real-time reactions to the threatening situation. Therefore, we focused on collecting the data in the exact timely context rather than on sample representativeness.

The indirect measurement of the variables—the intensity of negative emotions and the measure of threat due to the assessment of the situation in different places—requires comment. A peculiarity of indirect measures is that they are accompanied by a reasonable assumption about the mediation of certain processes (e.g., generalization). In the case of measuring emotions, we asked to what extent other people experience emotions. This solution was dictated by the desire to limit the potential negative affective experiences resulting from the analysis of one’s own negative emotions and to avoid the inevitable universal distortions derived from the dominance of the self [101]. On the other hand, this method of measurement was chosen to capture the actual experienced emotional intensity without taking into account defense mechanisms that might be associated with a tendency to report lower emotional intensity. Our research during the COVID-19 pandemic indicates that the declared intensity of perceived negative emotions is lower when emotions were asked about directly than when we used an indirect measure of emotion [77].

The use of one-item and two-item measures for the measurement of complex constructs may raise doubts. Nevertheless, such measurement tools are commonly employed in research on change, such as protective mechanisms [31,39,40,41]. Research on change demonstrates that concise measurements of this kind adequately fulfill their intended function.

Finally, the span of the scale measuring emotions changed between stages 0 and 1 due to changes in the platform used for the surveys. This change in the scale (from 1 to 100 to 1–7) limits the possibility of direct comparisons; therefore, we do not compare measurements from different stages with statistical analyses. The SEM models have been constructed separately for each stage, and the similarities and differences between the models are discussed qualitatively rather than analyzed statistically.

Regardless of the lack of alternative possibilities to conduct research in pandemic conditions, it is worth paying attention to the effects that digital tools and the digital context have on the quality of data collected and, consequently, on their interpretation and generalization of results [102,103]. Hence, irrespective of the objective possibilities of conducting the studies during the pandemic, it is worth paying attention to the effects that digital tools and the digital context have on the quality of data collected and, consequently, on their interpretation and generalization of results. The online procedure also limits the control and standardization of research conditions. The applied online procedures (one-time access to the questionnaire), the indirect nature of variable measurements, and a pilot study were used to increase the reliability of the responses.

Endnotes:

1. Variations on the emotional activity scale are regarded to be relatively independent from the type of an experienced emotion, its indication (positive or negative), or content [12,13].
2. Differences between the sense of danger at various distances have been described in a previous research article [77]. The study described in the article did not produce results on the intensity variation in the danger at various distances to the self and its relation to the other elements of the model. This confirms it is valid to treat the assessment of the situation in one’s own country, Europe, and the world as one of danger as an aggregated, single variable in the model.
3. In the research in Poland, Type 2 emotions still included embarrassment, which in the Ukrainian research was included in Type 1.
4. The identification of two factors among negative emotions in the context of questions about the dangers of the pandemic would seem to be related to processes of conscious self-regulation in two directions of attention: (self-focused attention, or (1) private self-consciousness, which serves to make one more aware of oneself, one’s own emotions, choices, and actions in self-regulation processes, and (2) public self-consciousness, which serves to monitor one’s own public presentation [104].

## Data Availability

The data presented in this study are openly available in FigShare at https://doi.org/10.6084/m9.figshare.21354180.v1, accessed on 12 March 2026.

## Supplementary Materials

The following supporting information can be downloaded at: https://www.mdpi.com/article/10.3390/ijerph23050606/s1, Supplementary Material S1: The pandemic situation in Poland and Ukraine during the study; Supplementary Material S2: Description of exploratory factor analyses and confirmatory analyses for the scales used in the study, accompanied by tables presenting descriptive statistics for the scales used in the study; Supplementary Material S3: The exact questions used in the study—English translation; Supplementary Material S4: Descriptive Statistics.
