# Supplementary material for: Selected Aspects of Self-Regulation: How People Cope with Danger and Change in the Context of COVID-19 (Research in Poland and Ukraine)
Source: Int J Environ Res Public Health. 2026 May 4;23(5):606. doi: 10.3390/ijerph23050606 (PMC13205806; doi:10.3390/ijerph23050606)
Supplement: Supplementary file 1 [file ijerph-23-00606-s001.zip › Supplementary_Material_S1.docx.pdf]

Supplementary Material S1

The pandemic situation in Poland and Ukraine during the study

The initial stage of this study reflected a period of high uncertainty and alarm, characterized by rapid institutional closures, strict containment measures, and intense media attention. Subsequent stages captured a shift toward adaptation and normalization, with rising case numbers accompanied by eased restrictions, declining public concern, and lower compliance with preventive measures. The exact description of the situation in Poland when this study was conducted can be found in our previous article [81].

As for the situation in Ukraine, the Ukrainian government declared a nationwide COVID-19 pandemic quarantine on March 11th 2020. On March 12th, 43 suspected cases of COVID-19 were reported, with one confirmed case. On 16th March, new quarantine measures were announced, including the closure of cultural, shopping and entertainment centers as well as the suspension of intercity and interregional transportation services. Restaurants and cafés were only allowed to serve food for take-out or delivery, and mass events were prohibited. Grocery stores, gas stations, pharmacies, banking institutions, and insurance companies remained open.

On 25th March, the government implemented a nationwide emergency situation regime, which was later extended to 22nd May. During this period, educational institutions were closed, public gatherings were prohibited, and people over the age of 60 were advised to self-isolate. Public catering, shopping centers, and entertainment venues were closed as well as most shops. Air, rail, and bus passenger services were also suspended. On 2nd April, the government approved updated Resolution No. 211, which extended the quarantine period and further prohibited activities such as group gatherings, visiting public places without a face mask or respirator and visiting parks and sports playgrounds. Rules for home isolation and observation were also established. As of 3rd April, Ukraine confirmed 1,225 coronavirus cases and 20 deaths. By April 29, there were 9,866 confirmed cases and 261 deaths in the country.
