# Supplementary material for: Selected Aspects of Self-Regulation: How People Cope with Danger and Change in the Context of COVID-19 (Research in Poland and Ukraine)
Source: Int J Environ Res Public Health. 2026 May 4;23(5):606. doi: 10.3390/ijerph23050606 (PMC13205806; doi:10.3390/ijerph23050606)
Supplement: Supplementary file 1 [file ijerph-23-00606-s001.zip › Supplementary_Material_S2.docx.pdf]

Supplementary Material S2

Description of exploratory factor analyses and confirmatory analyses for the scales used in the study, accompanied by tables presenting descriptive statistics for the scales used in the study.

1. Emotions

We decided to run an exploratory factor analysis with Varimax rotation on the measurements of emotions. We decided on conducting the analysis with orthogonal rotation (expecting to achieve uncorrelated factors) to maximize the simplicity of the obtained solution. The factor analysis run on the data from Stage 0 identified two distinct factors, including following emotions: Factor 1: sorrow, helplessness, sadness, breakdown, depression, frustration, terror, suffering, disappointment, disillusionment and bitterness (11 emotions); Factor 2: contempt, disgust, aversion, abhorrence, repulsion, shame, embarrassment and envy (8 emotions). Humiliation was excluded from further analyses, as it was not included in any of the factors (having loadings of .02 and .041 in respectively first and second factor).

We calculated Cronbach's alphas for both scales of emotions in each of the stages, presented in Table 1. All the Cronbach's alpha had the value above .85. Additionally, we calculated differences between these two scales in each of the stages; the results are presented in Table 2. The intensity of emotions from Factor 1 was higher than those from Factor 2 in all stages of the study, Cohen's d differing from .55 to 1.48 (although it's important to note that these Cohen's d statistics should be considered as an internal indices only, as the scales may slightly differ between populations).

Table S1. Cronbach's Alphas for both emotional factors in all stages of the study.

Stage	Factor 1	Factor 2
Poland 0	.87	.88
Poland 1	.95	.94
Poland 2	.97	.96
Ukraine	.94	.90

Table S2. Summaries of statistics for t-tests comparing means from emotional factors 1 and 2 in all stages of the study.

Stage	Factor 1		Factor 2		t	p	Cohen's d [CI 95%]
	M	SD	M	SD			
Poland 0	70.43	17.59	36.92	21.54	51.24	< .001	1.48 [1.40 – 1.56]
Poland 1	4.60	1.31	3.65	1.37	29.71	< .001	.87 [.80 - .93]
Poland 2	4.23	1.43	3.43	1.41	24.35	< .001	.77 [.69 - .83]
Ukraine	3.18	1.53	2.16	1.27	26.10	< .001	.82 [.75 - .90]

2. **Protective mechanisms: positive future Self and goal congruence**

Correlation between the two questions used for measuring the protective mechanisms were: $r = .62$, $p < .001$ for Stage 0, $r = .70$, $p < .001$ for Stage 1, $r = .77$, $p < .001$ for Stage 2 and $r = .43$, $p < .001$ for the measurement conducted in Ukraine.

3. **Sense of danger: individual sense of danger and the sense of danger at various distances**

The three questions about the perceived danger were aggregated because of their strong multicollinearity (VIF > 5 and tolerance factor below 0.1). Cronbach's Alphas for both variables for each of the stages are presented in Table 3.

Table S3. Cronbach's Alphas for both measures of sense of danger in all stages of the study.

Stage	Individual sense of danger	Sense of danger at various distances
Poland 0	.78	.90
Poland 1	.89	.91
Poland 2	.89	.91
Ukraine	.79	.87
