# Supplementary material for: Selected Aspects of Self-Regulation: How People Cope with Danger and Change in the Context of COVID-19 (Research in Poland and Ukraine)
Source: Int J Environ Res Public Health. 2026 May 4;23(5):606. doi: 10.3390/ijerph23050606 (PMC13205806; doi:10.3390/ijerph23050606)
Supplement: Supplementary file 1 [file ijerph-23-00606-s001.zip › Supplementary_Material_S3.docx.pdf]

Supplementary Material S3

The exact questions used in the study - English translation.

1. Assess to what extent emotions listed below cause people to experience a sense of threat in the current situation:

Suffering

To a small extent — To a high extent

1——————————————————100 (7 in stages 1 and 2)

Helplessness

To a small extent — To a high extent

1——————————————————100

Frustration

To a small extent — To a high extent

1——————————————————100

Breakdown

To a small extent — To a high extent

1——————————————————100

Terror

To a small extent — To a high extent

1——————————————————100

Bitterness

To a small extent — To a high extent

1——————————————————100

Aversion

To a small extent To a high extent

1———————————————————————100

Disgust

To a small extent To a high extent

1———————————————————————100

Abhorrence

To a small extent To a high extent

1———————————————————————100

Repulsion

To a small extent To a high extent

1———————————————————————100

Humiliation

To a small extent To a high extent

1———————————————————————100

Shame

To a small extent To a high extent

1———————————————————————100

Embarassment

To a small extent To a high extent

1———————————————————————100

Disappointment

To a small extent To a high extent

1———————————————————————100

Disillusionment

To a small extent To a high extent

1————————————————————————100

Sadness

To a small extent To a high extent

1————————————————————————100

Sorrow

To a small extent To a high extent

1————————————————————————100

Depression

To a small extent To a high extent

1————————————————————————100

Envy

To a small extent To a high extent

1————————————————————————100

Disdain

To a small extent To a high extent

1————————————————————————100

2. To what extent are you able to imagine yourself and your positive future?

I am not able at all I am definitely able to

1———2———3———4———5———6———7

3. To what extent are you able to achieve your everyday work and personal goals now?

I am not able at all I am definitely able to

1————2————3————4————5————6————7

4. To what extent are you able to achieve goals and values important for you, that originate from before the epidemic?

I am not able at all 		I am definitely able to

1————2————3————4————5————6————7

5. Considering the current situation, to what extent do you feel threatened physically (by pain, afflictions, disease)?

I do not feel worried at all 		I definitely feel worried

1————2————3————4————5————6————7

6. To what extent do you feel threatened mentally (by worries, anxiety, fear, panic)?

I do not feel worried at all 		I definitely feel worried

1————2————3————4————5————6————7

7. To what extent do you feel threatened by the dynamics of the epidemic (by getting infected)?

I do not feel worried at all 		I definitely feel worried

1————2————3————4————5————6————7

8. To what extent do you feel threatened by the epidemic situation in Poland?

To a small extent 		To a high extent

1————2————3————4————5————6————7

To what extent do you feel threatened by the epidemic situation in Europe?

To a small extent 		To a high extent

1————2————3————4————5————6————7

To what extent do you feel threatened by the epidemic situation in the whole world?

To a small extent To a high extent

1————2————3————4————5————6————7

10. Have you contracted the COVID-19 disease?

Stage 1: YES/NO

Stages 2, 3 and 4:

- yes, confirmed with a test
- probably yes, but I haven't had a test
- yes I have, which was confirmed with a test, I have already recovered
- probably yes, but I haven't had a test, I have already recovered
- I haven't contracted the COVID-19 disease
- I don't know

Has someone you know contracted the COVID-19 disease? (Stage 1 only)

YES/NO

Please indicate your sex:

- Woman
- Man
- Other (Stage 1 only)

Please indicate your age (in years):

…
