# Supplementary material for: Selected Aspects of Self-Regulation: How People Cope with Danger and Change in the Context of COVID-19 (Research in Poland and Ukraine)
Source: Int J Environ Res Public Health. 2026 May 4;23(5):606. doi: 10.3390/ijerph23050606 (PMC13205806; doi:10.3390/ijerph23050606)
Supplement: Supplementary file 1 [file ijerph-23-00606-s001.zip › Supplementary_Material_S4.docx.pdf]

Supplementary Materials S4 – Descriptive Statistics

Table S4. Descriptive statistics for the Stage 0 study (Poland).

Variable	*M*	*SD*	*Min*	*Max*
Emotions Factor 1	70.43	17.59	0	100
Emotions Factor 2	36.92	21.54	0	100
Positive future Self	2.11	1.75	1	7
Goal congruence	4.18	1.14	1	7
Individual sense of danger	4.72	1.57	1	7
Sense of danger at various distances	5.12	1.67	1	7

Table S5. Descriptive statistics for the Polish database (Stage 1).

Variable	*M*	*SD*	*Min*	*Max*
Emotions Factor 1	4.60	1.31	1	7
Emotions Factor 2	3.65	1.37	1	7
Positive future Self	4.38	1.46	1	7
Goal congruence	4.21	1.40	1	7
Individual sense of danger	4.02	1.60	1	7
Sense of danger at various distances	4.47	1.40	1	7

Table S6. Descriptive statistics for the Polish database (Stage 2).

Variable	*M*	*SD*	*Min*	*Max*
Emotions 1	4.23.	1.43	1	7
Emotions 2	3.40	1.41	1	7
Positive future Self	4.42	1.61	1	7
Goal congruence	3.14	1.22	1	7
Individual sense of danger	3.10	1.40	1	7
Sense of danger at various distances	4.47	1.24	1	7

Table S7. Descriptive statistics for the Ukrainian sample.

Variable	*M*	*SD*	*Min*	*Max*
Emotions Factor 1	3.18	1.53	1	7
Emotions Factor 2	2.16	1.27	1	7
Positive future Self	3.21	1.88	1	7
Goal congruence	3.09	1.41	1	7
Individual sense of danger	2.62	1.57	1	7
Sense of danger at various distances	4.27	1.57	1	7
